# Nano Emulsion of Essential Oils Loaded in Chitosan Coating for Controlling Anthracnose in Tomatoes (*Solanum lycopersicum*) During Storage

**DOI:** 10.3390/foods14173038

**Published:** 2025-08-29

**Authors:** Sibahle Gumede, Semakaleng Mpai, Sreejarani Kesavan Pillai, Dharini Sivakumar

**Affiliations:** 1 Phytochemical Food Network Group, Department of Crop Sciences, Tshwane University of Technology, Pretoria West 0001, South Africa; gumedesbahle95@gmail.com (S.G.); mpais@tut.ac.za (S.M.); 2 Centre for Nanostructures and Advanced Materials, DSI/CSIR Nanotechnology Innovation Centre, Council for Scientific and Industrial Research, Pretoria 0001, South Africa; skpillai@csir.co.za; 3 Centre for Nutrition & Food Sciences, Queensland Alliance for Agriculture and Food Innovation, The University of Queensland, Brisbane, QLD 4108, Australia

**Keywords:** *Colletotrichum gloeosporioides*, edible coating, nano emulsion, postharvest quality, thyme oil

## Abstract

Tomato fruit is susceptible to decay caused by *Colletotrichum gloeosporioides.* An edible coating derived from essential oils loaded into a chitosan polysaccharide polymer is a sustainable delivery approach to improve coating versatility and stability for reduced reliance on synthetic fungicides to combat anthracnose incidence in tomatoes. The objective of this study was to evaluate the antifungal efficacy of nanostructured thyme essential oil incorporated into chitosan coatings [Nano-(T)-EO-CS] against *Colletotrichum gloeosporioides* in tomato fruits, and to investigate the underlying mechanisms contributing to its inhibitory effects. Nano-(T)-EO of (1% *v/v*) showed the greatest antifungal activities while achieving complete inhibition of *C*. *gloeosporioides*. At (0.8% *w/v*) concentration, chitosan inhibited 78% of radial mycelial growth in *C. gloeosporioides*. Loading Nano-(T)-EO (1% *v/v*) into chitosan (0.8% *w/v*) completely inhibited spore germination (100%). The surface electron microscopy revealed that the Nano-(T)-EO-CS coating induced significant deformation and inhibited the growth of *C. gloeosporioides.* Compared with the control, the Nano-(T)-EO-CS coating reduced disease incidence by 50%, whereas the commercial antifungal agent Sporekill^®^ reduced incidence by 40% in preventively inoculated tomatoes stored at 10 °C and 85% relative humidity (RH) for 14 days after harvest, and at 18 °C for 3 days at the market shelf condition. Despite chitinase activity peaking on day 14 of cold storage, it peaked significantly on day 7 in Nano-(T)-EO-CS and Sporekill^®^-treated tomatoes. *The* Nano-(T)-EO-CS coating enhanced ferric-reducing antioxidant power and total phenol content in tomatoes for 7 and 14 d of postharvest storage. The chitosan-based edible coating loaded with thyme essential oil offers a sustainable, eco-friendly alternative to chemical fungicides for improving tomato shelf life and reducing decay.

## 1. Introduction

Tomatoes (*Solanum lycopersicum*), belonging to the family *Solanaceae*, are an economically important fruit with a high nutritional value. Global tomato production is estimated to be around 321 million tonnes in 2023, according to FAO data [1]. However, tomatoes have a short shelf life of 5 to 7 days due to their high respiration rate, sensitivity to ethylene, temperature fluctuations, relative humidity, and microbial degradation [2]. In tomato fruit, anthracnose rot caused by *Colletotrichum gloeosporioides* [3] is a common postharvest disease during ripening and storage. To control such postharvest pathogens, Sporekill^®^ (0.1% *v/v* solution, containing didecyl-dimethylammonium chloride) has been widely used in tomato packhouses as a broad-spectrum disinfectant for dump tanks. However, this quaternary ammonium compound poses environmental and health concerns, including toxicity to aquatic organisms, skin sensitivity, respiratory irritation, and reduced efficacy in the presence of organic matter [4]. Furthermore, didecyl-dimethylammonium chloride was revoked and replaced by the EU Directive 2009/128/EC Commission regulation on sustainable pesticide use on 22 June 2022, and it can no longer be applied for the prevention of microbial infections in fresh produce (including tomatoes). In this regard, there is a necessity to investigate alternative strategies to combat *C. gloeosporioides* diseases.

A great deal of interest has lately developed in the use of EOs as potential non-toxic substitutes for traditional synthetic fungicides [5]. It is possible that EOs’ components have no residual effect or a very low residual effect and may act as biofungicides [6]. The Food and Drug Administration (FDA) has labelled EOs as “Generally Recognised as Safe” (GRAS), meaning they are safe [7]. EOs’ antifungal properties are likely attributed to the presence of terpenes/terpenoids, which are lipophilic and have low molecular weight, which enables them to break down the membrane, causing cell death, or suppressing the sporulation and germination of decay-causing fungi [8]. A variety of factors limit the application of EOs, including their low water solubility, flammability, and physical and chemical instability, along with their effects on the sensory properties of fruits [9].

In food industries, nano emulsions with oil-in-water emulsions are providing more potential for expansion by transitioning to a high-pressure homogenisation process that can be readily combined with edible ingredients [10]. In these nano emulsions, the nano-metric drops (sizes below 100 nm) are suspended in an aqueous continuum phase and covered with a film or layer of food-grade constituents [10]. Nano emulsions can be formulated as edible coatings for fruits, such as papaya, mango, and strawberries, improving the dispersion of active chemicals and protecting them from decay [10].

The deacetylation of chitin produces the cationic polysaccharide chitosan (CS). Chitosan is growing in use due to its low toxicity, antimicrobial and antibacterial properties, and coating-forming abilities that make it economically feasible [11]. Since essential oils are lipophilic, they can alter the water solubility and water vapor permeability of CS coatings, slowing fruit ripening and respiration rates [12]. For example, applications of chitosan (1% *w/v*) and thyme oil (400 μL/L) prevented the in vitro mycelial growth of *C. gloeosporioides* from mango fruit, and this was associated with the antifungal activity of the thymol volatile compounds contained [13]. Regarding tomato fruit, some studies highlighted that chitosan concomitant with essential oils such as *Ruta graveolens* and *Schinus molle* presented a growth inhibition of some fungi pathogens including *C. gloeosporioides* and *Fusarium oxysporum* [14]. However, it is critical to optimize the concentrations of both the chitosan and essential oil on individual phytopathogens, especially since they may excrete different pH levels which contribute to their response to these edible coatings.

Therefore, the purpose of this study was to determine the effectiveness of nanostructured chitosan–thyme oil coatings in controlling *C. gloeosporioides* considering in vitro and in vivo trials in tomato fruits and underpinning the mode of action during postharvest storage.

## 2. Materials and Methods

### 2.1. Chemicals

The chitosan (CS, MW 50–190 kDa, deacetylation degree 75–85%, purity 75%) and all other chemicals were procured from Sigma Aldrich (St. Louis, MO, USA). Holistic Emporium (Edenvale/Gauteng 1612, South Africa) provided 100% thyme (T), *Thymus* L., lemongrass (L) (*Cymbopogon nardus*), and sage (*Salvia officinalis*) (S) essential oils (EOs). Sporekill^®^ commercial disinfectant was bought from Hygrotech (ICA International Chemicals (Pty) Ltd., Pretoria North, South Africa).

### 2.2. Fungal Cultures

Tshwane University of Technology’s Fruit and Vegetable Technology Laboratories provided fungi cultures (*Colletotrichum gloeosporioides*). The DNA sequenced original fungal cultures were obtained from ARC-PHP, Roodeplaat (West), Pretoria, South Africa. The fungal isolates were maintained at 25 °C for 12–13 days on potato dextrose agar (PDA). On PDA plates, fungal isolates were subcultured to obtain a pure and distinct colony.

### 2.3. Inhibition Rate of EOs and CS by the Contact Method

A poison food technique was used to evaluate the efficacy of (T)-EO or and (L)-EO and (S)-EO to inhibit radial mycelial growth of tomato fungus decay [15]. EOs from (T), (L), and (S) were tested against *C. gloeosporioides* for concentrations of 0.25%, 0.5%, 1%,1.5%, 2%, 2.5%, 3.0%, 3.5%, and 4% *v/v*. Chitosan coating concentrations were screened from 0.2%, 04%, 0.6%, 0.8%, and 1% *w/v*. To attain the specific final concentration of (T)-EO, (L)-EO and (S)-EO in PDA media in the Petri dishes, the required volume of (T)-EO, (L)-EO and (S)-EOs was dissolved in 1 mL of 0.5% Tween 80 (*v/v*) with sterile distilled water (DW), and then transferred into PDA media at (37–40 °C) under sterile conditions (Javanmardi et al. 2023) [9].To prepare the CS solution, 0.2, 0.4, 0.6, and 0.8 g of CS were mixed sequentially with 100 mL of purified water and 0.5 mL of acetic acid. The solutions were continuously stirred using a magnetic stirrer (Hotplate Stirrer, IKA^®^ C-MAG HS 7, IKA Werke GmbH & Co. KG, Staufen, Germany) at 120 rpm for 24 h at room temperature (25 °C) to ensure the complete dissolution of chitosan. The initial pH of the chitosan solution was 3.5 and 1 N NaOH was injected to maintain a pH of 5.6, as shown by Maswanganye et al. [15]. The above method was used to test the inhibitory effects of different concentrations of CS on *C. gloeosporioides* in vitro. As controls, negative control plates consisted of PDA supplemented with 0.5% Tween 80 (*v/v*) (without EOs or chitosan), while the positive control consisted of PDA amended with Sporekill^®^ at 1.5% (*v/v*), a commercial disinfectant. Five-day-old mycelia discs of *C. gloeosporioides* with a diameter of 5 mm were removed using a sterile cork borer and placed in sterile Petri dishes containing PDA + T-EOs, PDA + L-EOs, PDA + S-EOs, and CS + PDA. (0.2%, 0.4%, 0.6%, 0.8% *w/v*), with three replicates per treatment. To prevent volatile EOs from escaping, two layers of parafilm were used to cover the Petri dishes.

After incubation at 25 °C for 7 days, the radial growth of the fungus was recorded every day until it filled the margins of the control dish. Colony diameter was recorded using Vernier callipers (Model No. 500-196-30, Caliper Digimatic, Mitutoyo Co., Kawasaki, Kanagawa, Japan). Antifungal activity was assessed using the formula below [15] as a percentage of Inhibition of Mycelia Growth (IMG).

$$Inhibition\;of\;mycelial\;growth\left(\%\right)=\frac{dc-dt}{dc}\times 100$$

dc = average diameter of control plate, dt = average diameter of treated plate.

The minimum inhibitory concentrations (MICs) of the antifungal agents were defined as the lowest concentrations that prevented visible growth of the microorganism, as indicated by the absence of colony diameter increase on PDA plates after incubation. [16]. The MICs play a crucial role in determining an organism’s antifungal susceptibility [16]. The mycelial plugs that displayed no growth were deposited on freshly poured PDA plates to evaluate the fungicidal or fungistatic activity of Nano (T)-EO and CS coating. After 7 days of incubation at 25 °C, fungal growth and recovery was monitored. To assess fungal recovery, a 5 mm mycelial disc was aseptically taken from the centre of treated plates that showed no visible growth and transferred onto fresh PDA plates without any antifungal agents. The plates were then incubated at 25 °C for 5 to 7 days to observe whether fungal growth resumed, indicating whether the treatment was fungistatic or fungicidal. A fungicidal effect is characterized by no growth, whereas a fungistatic effect is characterized by growth.

### 2.4. The Effect of Nano-EO and CS on the Growth of C. gloeosporioides In Vitro

#### Development of Nano-(T)-EO

Nano-(T)-EO was prepared according to Elshamy et al. [17] without any modifications using a continuous phase initially containing phosphate pH standard equimolar solution at pH 6.68, modified lecithin (0.1% *w/w*), and Tween 80 (2% *v/v*). Soybean oil (1% *v/v*) and thyme oil (1% *v/v*) comprised the dispersed phase. Homogenisation of the O/W mixture was accomplished using an IKA^®^ T25 Digital Ultra-Turrax^®^ homogeniser (IKA-Werke GmbH & Co. KG, Staufen, BW, Germany), (10,000 rpm for 10 min) with subsequent ultrasonication at 20 °C for 10 min using the Skymen Ultrasonic JP 100S (Skymen Cleaning Equipment Co. Ltd., Shenzhen, China) and followed by a high-pressure homogeniser at 100 MPa for two cycles to obtain a stable and uniform nano emulsion with reduced particle size and improved physical stability. The experiments were designed so that the final Nano emulsion had (T)-EOs with concentrations of 1% based on Section 2.3. The phase difference index (PDI) of Nano-EMs was evaluated using dynamic light scattering (DLS) using Zetasizer (Malvern Zetasizer Nanosizer^®^, Malvern Instruments Ltd., Worcestershire, UK) [15].

### 2.5. Development of CS and Nano (T)-EO Edible Coatings with Antifungal Properties

The 0.8% CS (*w/v*) was prepared first, followed by the previously prepared Nano-(T)-EO emulsion at 1% (*v/v*). The coating mixtures were stirred at room temperature for 10 min to ensure complete homogenisation and adjust the final concentrations of CS and emulsion. The antifungal properties of the developed CS and Nano-(T)-EO edible coatings were measured as mentioned above by assessing the percentage inhibition of radial mycelial growth as shown in Section 2.3.

### 2.6. Inhibition of Spore Germination 

Nano-(T)-EO emulsion and Nano-(T)-EO-CS coatings were evaluated for their ability to suppress spore germination as described by Sivakumar et al. [18]. After separating the spores from the mycelium and fragments of the solid PDA medium using double layers of cheesecloth, the spore concentration was calibrated with a hemocytometer to 10^6^ spores/mL. To determine the impact of postharvest treatments on spore germination, fresh spore suspensions of *C gloeosporioides* were added to potato dextrose broth supplemented with different concentrations of treatments and the cultures were held for 24 h at 28 °C. The experiment was replicated twice with three replicates. Using a microscope (Axiophot Carl Zeiss, Carl Zeiss Microscopy GmbH, Jena, Germany) at ×100 magnification, 6 random microscopic fields were examined per plate, and the percentage of germinated spores was calculated. The germination of 200 spores per plate was monitored after incubation. Conidia are considered germinated when their germ tubes reach half their length. Sterile distilled water (0.1 mL) and Sporekill^®^ (1.5% *v/v*) treatments stood as control. Standalone CS (0.8% *v/v*) and Nano-(T)-EO emulsion (0.25%, 0.5%, 1% *v/v*) Nano-(T)-EO (0.25% *v/v*)-CS, Nano-(T)-EO (0.5% *v/v*)-CS, and Nano-(T)-EO (1% *v/v*)-CS were included for comparison.

Each treatment was replicated three times. Based on the following formula, we estimated the inhibition rate (%) of spore germination of isolated *C. gloeosporioides:*

$$\%\;Inhibition\;of\;germination=\frac{N1-N2}{N1}\times 100$$

N1: Number of spores that germinate without treatment.

N2: Number of spores germinating in the presence of treatments. The IC_50_ values (concentration that would result in 50% inhibition of germination) were calculated from data on spore germination and calculated from nonlinear regression models [19].

### 2.7. In Vivo Antifungal Activity of Nano-(T)-EO-CS Coating Formulation 

**Figure 1:**
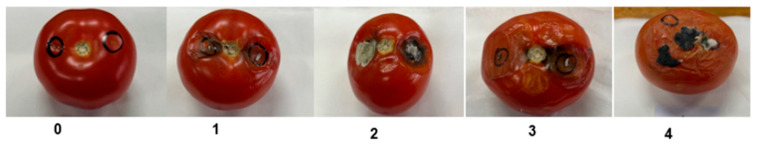
Visual scaling for determining the anthracnose decay severity in vivo inoculation.

Tomatoes (classic round variety) were harvested at commercial maturity without any postharvest treatments and sorted for uniform size and defect-free quality. Using a 0.01% (*v/v*) NaOCl solution for 1 min, tomato fruits were surface sterilized and air-dried for 20 min. In each fruit, a sterile needle (5 mm depth × 2 mm width) was inserted on opposing sides and wounded on the surface [20]. An inoculation of 20 µL (10^6^ spores/µL) of *C. gloeosporioides* spore suspension was administered separately into each wound. After inoculation, the fruits were incubated at 25 °C for 8 h to allow the wounds to dry and were dipped in Nano-(T)-EO (1% *v/v*)-CS (0.8% *w/v*) coating (treatments) or distilled water (control) and Sporekill ^®^ for 1 min and air-dried after incubation at 25 °C at 85% RH for 8 h. Three replicate boxes containing 15 fruits were used for this experiment. As a preventive treatment, Nano-(T)-EO (1% *v/v*)-CS (0.8% *w/v*) coating was applied to tomatoes by dipping, followed by 15 min air drying. Following application, the fruits were inoculated with *C. gloeosporioides conidial* suspension (10^6^ spores/mL) as described earlier. A total of five replicate boxes of 15 tomatoes per treatment were used for preventive trials. Uncoated and coated tomatoes were stored at 10 °C and 85% RH for 7 and 14 days, and then at 18 °C for up to 3 days to mimic the market shelf condition. Nano-(T)-EO (1% *v/v*)-CS (0.8% *w/v*) coating, Nano-(T) EO (1% *v/v*), Sporekill^®^ (1.5% *v/v*)-treated tomatoes, and untreated controls were included in this study. The decay severity of the rotten tomato was assessed based on the developed scale: 0 = no infection, 1 = mild infection (1–25%) of the area covered by slight necrotic and fungal mycelia), 2 = moderate infection (26–50%) of the surface area covered by necrotic and fungal mycelia), 3 = severe infection (51–75%) of the tomato is necrotic with spore masses), 4 = very severe (≥78% of necrotic tissue with fungal mass and the fruit appears soft and decayed) as shown in Figure 1.

### 2.8. Scanning Electron Microscopy (SEM)

For scanning electron microscope (SEM) observation, the mycelia and spores were fixed with 2.5% (*v/v*) glutaraldehyde in 0.1 M phosphate buffer (pH 7.2) at 4 °C for 3 h, and then further dehydrated in a graded ethanol series (25%, 50%, 70%, 95%, and 100% *v/v* three times), critical-point-dried and mounted on specimen stubs, then coated with gold using a Quorum auto fine coater (Quorum, Q150T Auto Fine Coater (Quorum Technologies Ltd., Laughton, East Sussex, UK) England), and examined with a scanning electron microscope (Zeiss Ultra-Plus FEG-SEM, Carl Zeiss Microscopy GmbH, Oberkochen, BW, Germany) [21].

### 2.9. Defence Response and Antioxidant Activity

The enzyme assay, chitinase activity, and antioxidant activity (ferric-reducing antioxidant power) were also carried out on inoculated fruit coated with Nano (T)-EO (1%)-CS (0.8%) coating (preventive treatment). The activity of the chitinase enzyme was determined according to Sellamuthu et al. [22]. Tissue samples (1 g) were collected 2 mm away from the lesion site without peeling the fruits to accurately assess the local biochemical responses to infection and treatment. The enzyme extract (600 µL) and 2% (*w/v*) dye-labelled chitin azure (125 μL) were incubated for 2 h at 40 °C in 50 mM sodium acetate buffer (pH 5.0). An aliquot of 5 μL of 1 M HCl was added at the end of the reaction. At 550 nm, one unit was defined as the amount of enzyme needed to catalyse the formation of 1 nmol product h^−1^ mg of protein^−1^. Chitinase activity was measured for Nano (T)-EO (1%)-CS (0.8%) coating, Nano-(T) EO (1%), Sporekill^®^-treated tomatoes, and untreated controls stored at 0, 7, and 14 days.

The ferric-reducing antioxidant power (FRAP) was assessed using the method outlined by Xiao et al. [23]. Antioxidant activity was measured by mixing 75 μL of sample extract with 1425 μL of FRAP working solution, which consisted of 300 mM/L acetate buffer (pH 3.6), 10 mM/L TPTZ (2,4,6-tris(2-pyridyl)-1,3,5-triazine), and 20 mM/L ferric chloride. The mixture was incubated in the dark for 30 min, and absorbance was recorded at 593 nm. Results were expressed as mM Trolox equivalents (TE) per gram of dry weight (g/DW).

### 2.10. Statistical Analysis

Statistical analysis was carried out using GenStat 64-bit version 22.1 (Hempstead, England). The treatments were laid out in a completely randomized design. The experiment was repeated twice with 10 replicates for all in vitro studies. For the in vivo experiments, three replicate boxes each containing 13 fruits were used for each treatment. All in vitro experiments were evaluated using one-way analysis of variance (ANOVA), and results are presented as mean ± standard deviation (SD). Two-way ANOVA was used in the in vivo trial to evaluate the effectiveness of the treatments with storage time (14 and 21 days). Fisher’s protected test was used to identify significant differences between mean values at *p* < 0.05.

## 3. Results and Discussions

### 3.1. Nano (T)-EO-CS on Inhibition Rate of C. gloeosporioides Mycelia

**Figure 2:**
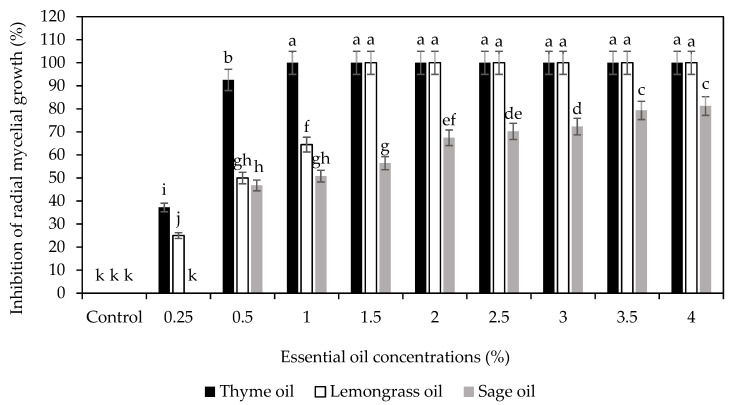
Screening of thyme, lemon, and sage essential oil nano emulsions at different concentrations against the mycelial growth (mm) of *C. gloeosporioides* after 7 days of incubation at 25 °C. Bars with different alphabet letters in the same column are significantly different (*p* < 0.05).

Using antifungal activity assays, it was found that the inhibitory effect of EOs on *C. gloeosporioides* increased with an increase in concentration, but the effectiveness of the EOs on inhibition of *C. gloeosporioides* radial mycelial growth followed the order (T)-EO>(L)-EO>(S)-EO (Figure 2). Sellamuthu et al. [22] observed that thyme essential oil exhibited the highest radical mycelia growth inhibition compared with citronella and peppermint essential oils, owing to the thymol, a key component of the oil known for its antimicrobial and antifungal properties. Maswanganyi [15] showed that the nano-encapsulated 2% (*v/v*) spearmint essential oil (SEO) loaded onto CS (0.8% *w/v*) inhibited the radial mycelial growth and spore germination growth of *Penicillium italicum* and *Penicillium digitatum* in an equal manner to the positive chemical fungicide (Imazalil) and better than individual coating components. Moreover, a complete radical mycelia growth of C. *gloeosporioides* was detected by the application of combined CS (1% *w/v*) and thyme oil (1% *v/v*) in a 3:1 (*v/v*) coating [20], which supports the current results on its effectiveness on in vitro trials. This suggests the potential effect of different EOs and CS as stable edible coating combination against different fruit pathogens.

**Figure 3:**
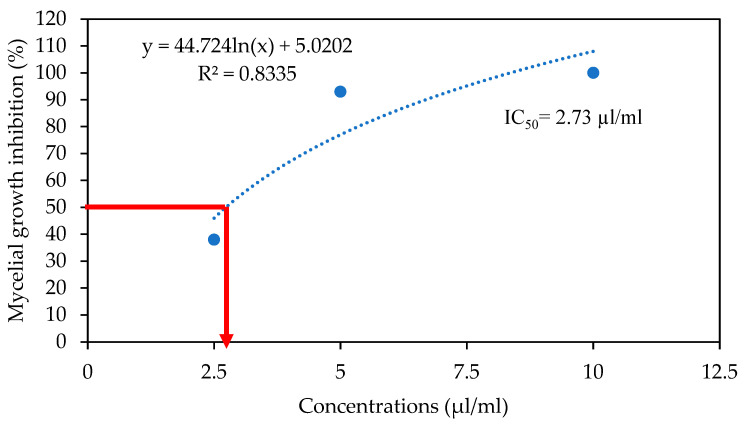
Nonlinear regression graph for mycelial growth inhibition (%) of *Colletotrichum gloeosporioides* on PDA Petri dishes enriched with thyme oil at different concentrations after 7 days incubation at 25 °C in vitro.

**Figure 4:**
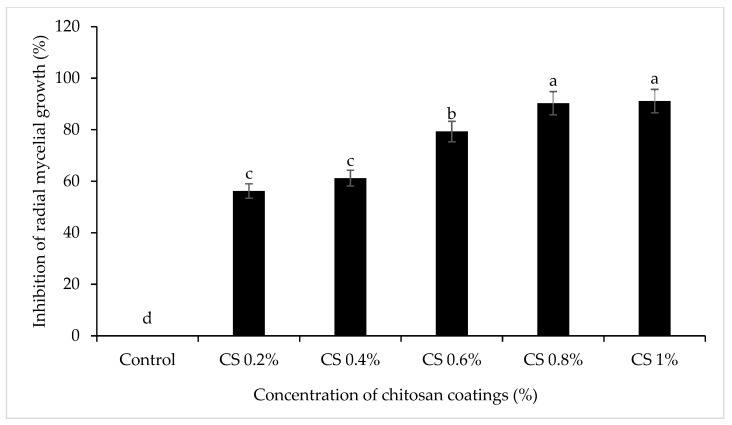
Screening of different concentrations of chitosan coating against the mycelial growth (mm) of *C. gloeosporioides* after 7 days of incubation at 25 °C. Bars with different alphabet letters in the same column are significantly different (*p* < 0.05).

The mycelial growth of *Colletotrichum gloeosporioides* on PDA plates supplemented with different concentrations of thyme oil was assessed after 7 days of incubation at 25 °C. Nonlinear regression analysis was used to establish the relationship between essential oil concentration and fungal growth inhibition. As shown in Figure 3, the red line indicates the IC_50_ value, calculated as 2.73 µL/mL, representing the concentration required to inhibit 50% of mycelial growth. The minimum inhibitory concentration (MIC) was determined to be 1% *(v/v*) T-EO, which achieved complete inhibition of *C. gloeosporioides*.. Additionally, at 0.8% (*w/v*) concentration, chitosan showed 78% radial mycelial growth inhibition of *C. gloeosporioides* (Figure 4). These results were similar to those reported towards the ability of different concentrations of chitosan to stop the proliferation in vitro of some fungi such as *C. gloeosporioides* [24,25].

Since the coating was viscous, chitosan concentrations were not increased, which could adversely affect tomatoes’ ripening and sensory characteristics. Due to its high inhibitory ability, (T-1% *v/v*)-EO was used to prepare the nano emulsion in the next experiment (in vivo study). (T)-EO contained the highest concentration of phenolic monoterpene thymol (58.77%) based on our previous investigation [18]. Nano-(T)-EO emulsion had a mean droplet size and PDI of 60.21 ± 1.8 nm. According to these results, the droplet size of prepared samples was comparable to that of nano emulsions [26]. Nano-(T)-EO and Nano-(T) EO-CS showed a fungistatic effect on in vitro radial mycelial growth inhibition while Sporekill^®^ completely inhibited the radial mycelial growth with fungicidal effect (Table 1).

**Figure 5:**
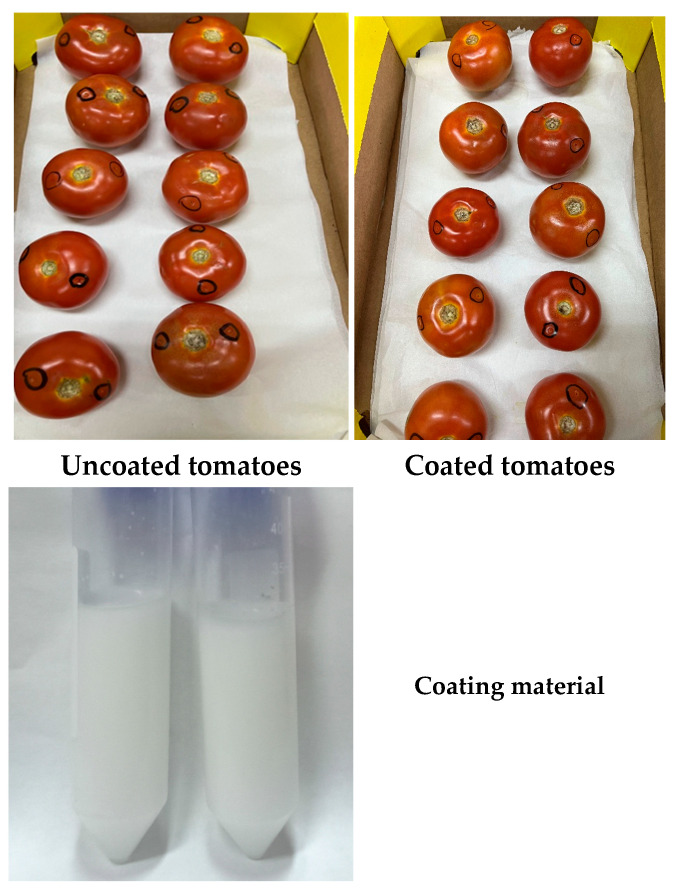
Visual appearance of the coating material and its effect on tomato surface characteristics; appearance of the nano emulsion chitosan–essential oil coating solution, showing its colour and transparency.

**Figure 6:**
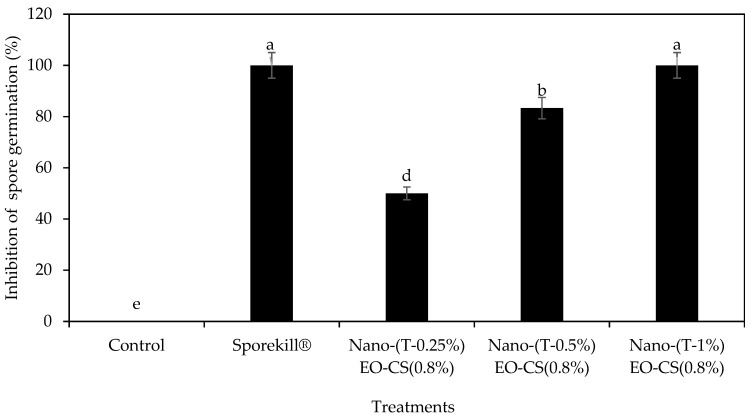
Chitosan coatings loaded with different thyme essential oil nano emulsions at different concentrations on the spore germination of *C. gloeosporioides* for 24 h at 25 °C. Nano-(T)-EO-CS coating loaded with thyme essential oil nano emulsion. Bars with different alphabet letters in the same column are significantly different (*p* < 0.05).

The visual characteristics of the coating material were evaluated prior to application. Figure 5 shows Nano-(T)-EO-CS coating solution was uniform in color and highly transparent, indicating a successful formulation. When applied to tomatoes, the coating formed a thin, even layer on the fruit surface, preserving its natural appearance while providing potential protective effects against postharvest spoilage. Figure 6 shows the impact of Nano-(T 1%)-EO-CS *coating* on the inhibition of spore germination of *C. gloeosporioides.* The MIC for spore germination for Nano-(T)-EO was 1.92 µL/mL, and the spore germination was completely inhibited at 1.5%. 

Higher concentrations of essential oil can cause shriveling of the fruit skin due to increased water loss [27]. Figure 6 shows the inhibition of spore germination in the chitosan (0.8%) coating loaded with different concentrations of Nano-(T)-EO emulsion on spore germination of *C. gloeosporioides* for 24 h. Nano-(T)-EO (1% *v/v*)-CS (0.8% *w/v*) demonstrated complete inhibition of spore germination Nano-(T)-EO-CS during 24 h incubation. Therefore, 1% Nano-(T)- EO was used in this study. Since most of the infection occurs via spores landing on fruit surfaces or entering through wounds, the effectiveness of the Nano-(T)- EO-CS coating was tested on the inhibition of spore germination of *C. gloeosporioides* [28]. Combining Nano-(T)- EO (1% *v/v*) with CS (0.8% *w/v*) enhanced the inhibition of spore germination to 100%.

**Figure 7:**
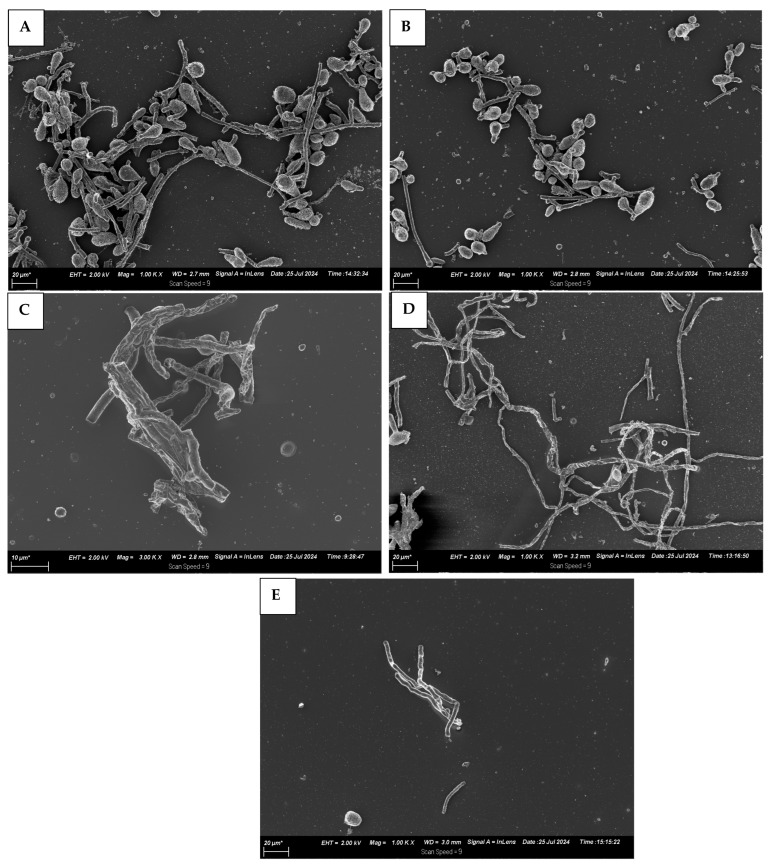
Scanning electron microscopy (SEM) images illustrating the hyphal morphology of *C. gloeosporioides* under different treatments: (**A**) control group (uncoated), (**B**) treated with 0.8% chitosan coating, (**C**) treated with 1% thyme nano emulsion, (**D**) treated with combination of 1% thyme nano emulsion and 0.8% chitosan, and (**E**) exposed to 1.5% Sporekill^®^. *: scale bar.

Based on SEM observations (Figure 7A–E), the control exhibited abundant mycelium and hyphae with smooth, uniform, and robust surfaces, along with well-defined conidia. In contrast, the T-EO treatment and the CS treatment resulted in significant deformation and inhibition of development. Additionally, the treated samples showed shrivelled, collapsed hyphae and deformed, irregularly shaped spores, indicating structural damage likely caused by the antifungal action of thyme nano emulsion coating. Spores appeared granular and swollen, the hyphae became fragile, opaque, and translucent, with irregular branches, culminating in the hyphae breaking. With Nano-(T)-EO-CS, the hyphae thin out and become empty, altering the structural appearance of the cell wall and impairing membrane permeability, allowing intracellular material to leak out [28]. Furthermore, chitosan interferes with various stages of fungal mycelium development, inhibiting its growth [29]. Recently, Maswanganye et al. [15] found uneven surfaces linked to CS coating embedded with spearmint essential oils, thus suggesting the impact of individual nano emulsion impact on the structural appearance of the coating.

The antimicrobial and antifungal properties of thymol are likely responsible for this effect. The hydrophobic nature of the oil’s chemical constituents also allows it to penetrate the lipid regions of microbial cell membranes [30]. It has been suggested that essential oils and their phytochemicals have antifungal properties [31]. In addition to disrupting cell membrane properties and impairing related functions, they thin and distort the wall of the hyphae, suppress glucan formation, disrupt fungal mitochondrial function, and inhibit efflux pumps. Chitosan may have antifungal properties due to its ability to damage the fungal cell membrane due to its amino group interactions with its phospholipids, which increases the membrane’s permeability [31]. The results obtained were similar to those from some previous work in which the microstructure of *A. niger* treated with essential oils was studied [32,33].

### 3.2. Nano-(T)-EO-CS Coating on the Inhibition of C. gloeosporioides in Inoculated Fruits

**Figure 8:**
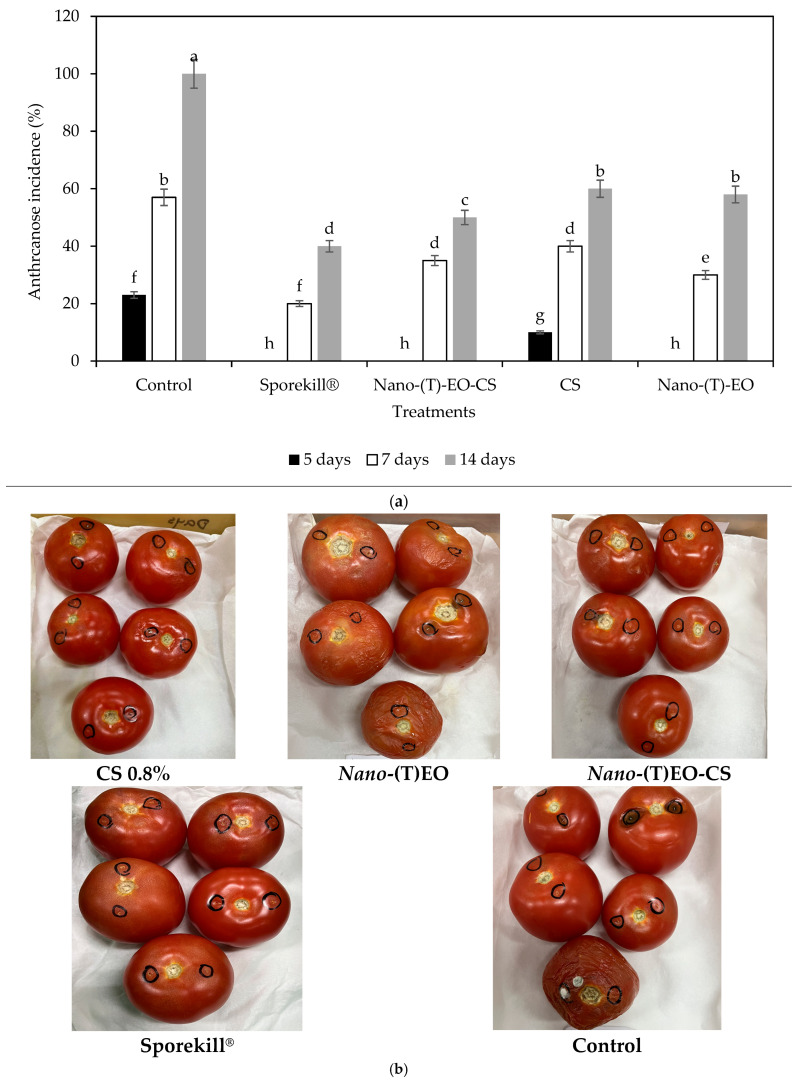
(**a**) Influence of chitosan coating loaded with thyme essential oil nano emulsion [Nano-(T) EO-CS] on anthracnose decay in preventively inoculated tomatoes after 5, 7, and 14 days of postharvest storage at 10 °C and 85% RH. Nano-(T) EO–thyme essential oil nano emulsion. Bars with different alphabet letters in the same column are significantly different (*p* < 0.05). (**b**) Anthracnose incidence and severity in preventatively inoculated tomatoes with *C. gloeosporioides*, stored at 10 °C and 85% RH for 14 days and 18 °C for 3 days at market shelf conditions.

Figure 8a,b illustrate anthracnose incidence and severity in artificially inoculated tomatoes with *C. gloeosporioides.* Visible symptoms appeared within three days after inoculation as shown in Figure 8b and increased significantly (*p* < 0.05) with prolonged storage. Untreated control fruits progressed at a significantly higher rate than those coated with Nano-(T)-EO-CS. After 14 days of cold storage, compared with the control, Nano-(T)-EO-CS coating reduced disease incidence by 50%, while Sporekill^®^ decreased it by 40% as illustrated in Figure 8a. On day 7 Nano-(T)-EO-CS coating and Sporekill^®^ reduced the anthracnose incidence to 20 and 35%, respectively, compared with the control. In comparison with previous studies, the application of chitosan combined with T-EO reduced the severity of *C. gloeosporioides* in mango fruit, with effects observed up to day 9. compared with individual coating components and the negative control [13]. The CS (1% *w/v*) combined with thyme oil (1% *v/v*) coating reduced the anthracnose (*C. gloeosporioides*) incidence and severity on avocado fruits more effectively than the commercially used prochloraz chemical fungicide [20]. This supports our results based on the effectiveness of the CS+T-EO combination over individual coating components on delaying the progression of infection from different phytopathogens, which promotes the shelf life of the produce. However, inconsistencies were observed depending on the storage duration and the effective concentrations of CS and essential oils used. Nano-(T)-EO-CS could be considered as an alternative treatment to replace Sporekill^®^. The decay severity was significantly reduced in Nano-(T)-EO-CS-coated fruits. These decayed fruits showed a decay severity score of 1 according to Figure 1. These results support the antifungal effects observed in the in vitro assays. The combination of chitosan (CS) and essential oils (EOs) has not always demonstrated the same level of antifungal efficacy in fruit, likely due to the high volatility of EO components and potential interactions between the coating materials and the fruit’s vegetative tissue [34]. The control of postharvest fungal diseases using chitosan (CS) coatings containing essential oils (EOs) appears to occur through both a direct inhibitory effect on fungal cells and an indirect effect by stimulating defence mechanisms in the fruit tissue [34]. It has been suggested that the effectiveness of CS-EO coatings in suppressing pathogenic fungi in fruit may be partially due to their ability to trigger the production of defence-related enzymes, such as polyphenoloxidase, peroxidase, chitinase, and β-1,3-glucanase, in the coated fruit [34].

CS coating incidence decreased by 40%, and Nano-(T)-EO alone by 30%. After 3 days of removal from cold storage, no anthracnose lesion severity was observed in CS, Nano-(T)-EO-CS, or Sporekill^®^-coated samples. However, Nano-(T)-EO-coated and control groups showed a similar severity of anthracnose. On the 5th day at 18 °C (after removal from cold storage), visible lesions began to appear on Nano-(T)-EO-CS and Sporekill^®^-coated fruit, though their severity was significantly lower than in the control group. Nano-(T)-EO-CS and Sporekill^®^ treatments consistently reduced anthracnose lesion severity (*p <* 0.05) compared with the control fruit and Nano-(T)-EO-treated fruits. The severity ratings for Nano-(T)-EO-CS and Sporekill^®^-coated fruits remained low at 1 rating score, while the control group showed a relatively higher severity rating of 4, followed by CS and Nano-(T)-EO at 2. Amoozegaran et al. [35] showed that the carboxymethyl cellulose coating containing thyme EO at 0.5%(*v*/*v*) concentration prevented Fusarium growth. *F*. *oxysporum* infection with 85.31% inhibition occurred in inoculated tomatoes after 9 days. Accordingly, in our study, we extended tomatoes’ shelf life to 14 days at the cold stage and 3 days under market shelf conditions by reducing the incidence of anthracnose.

### 3.3. Nano-(T)-EO-CS Coating on Chitinase Activity

**Figure 9:**
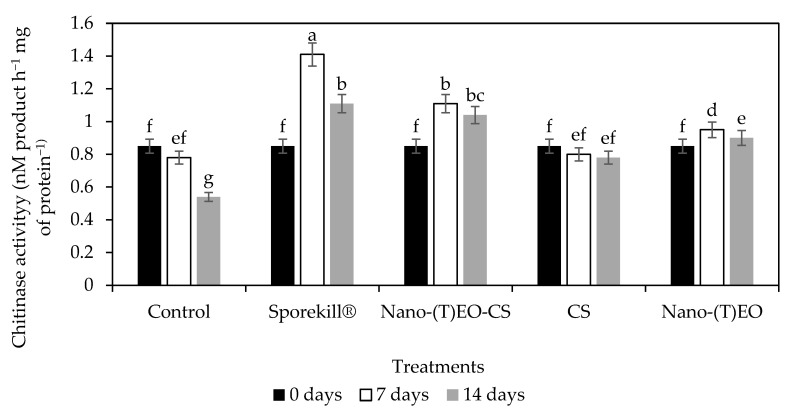
Influence of chitosan coating loaded with thyme essential oil nano emulsion [Nano-(T) EO-CS] coating on chitinase activity in tomatoes after 7 and 14 days of postharvest storage at 10 °C and 85% RH. Nano-(T)EO–thyme essential oil nano emulsion. Bars with different alphabet letters in the same column are significantly different (*p* < 0.05).

Pathogenesis-related proteins such as chitinase can facilitate the hydrolysis of the cell walls of fungal pathogens [36]. The change in chitinase activity during the infection process is illustrated in Figure 9. Chitinase activity was significantly higher in treatments than in the control. However, despite chitinase activity peaking on day 14 of cold storage, it peaked significantly on day 7 in Nano-(T)-EO-CS and Sporekill^®^-treated tomatoes. In addition to inhibiting fungal growth, these enzymes activate pathogen-borne elicitors that elicit subsequent defence responses in the fruit (host) [37].

### 3.4. Nano-(T)-EO-CS Coating on Total Phenols and Antioxidant Power

**Figure 10:**
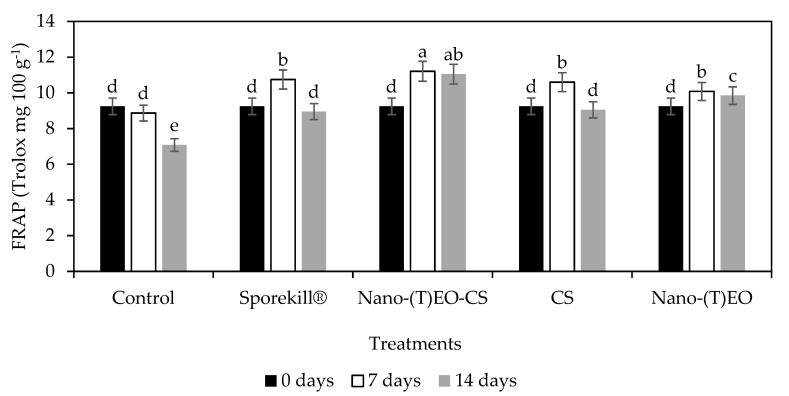
Influence of chitosan coating loaded with thyme essential oil nano emulsion [Nano-(T) EO-CS] on ferric-reducing antioxidant power in tomatoes after 7 and 14 days of postharvest storage at 10 °C and 85% RH. Nano-(T)EO–thyme essential oil nano emulsion. Bars with different alphabet letters in the same column are significantly different (*p* < 0.05).

The Nano-(T)-EO-CS coated fruits inoculated with *C. gloeosporioides* and held in cold storage for 7 and 14 days showed an increase in ferric-reducing antioxidant power (Figure 10), while phenolic compounds increased concurrently. The efficiency of phenolic compounds as antioxidants is largely dependent on their chemical structures, relative orientation, and number of hydroxyl groups attached to the aromatic ring [38]. Additionally, phenylpropanoid pathway enzymes, such as phenylalanine ammonia-lyase (PAL), might have been activated by infection and Nano-(T)-EO-CS coatings [39]. Moreover, by promoting higher PAL activity, which is an important factor in fruit disease resistance, chitosan in combination with thyme oil vapour application prevented the degradation of secondary metabolites (phenolic compounds or flavonoids) in avocados [20].

## 4. Conclusions

The development of a nano emulsion thyme essential oil incorporated into chitosan coatings [Nano-(T)-EO-CS] was developed to combat anthracnose disease in tomato fruit. The application of 1% thyme essential oil exhibited 100% mycelia growth inhibition, contributing to the minimum volume and affordable practical implications when compared with the 1.5% achieved in sage and lemongrass essential oils. Nano-(T)-EO (1% *v/v*)-CS (0.8% *w/v*) demonstrated enhanced matrix action on antifungal activity, inhibited anthracnose decay caused by *C. gloeosporioides*, and protected tomato fruits. This antifungal activity of Nano-(T)-EO-CS was supported by the surface electron microscopy structures showing an induced significant deformation of the growth of *C. gloeosporioides.*

Nano-(T)-EO-CS coatings, therefore, could provide an eco-friendly alternative to chemical synthesized Sporekill^®^, which contributes to the reduction of environmental impact using natural, biodegradable, and safe products for preserving the quality of tomatoes during postharvest storage. Moreover, this novel green antimicrobial agent can be used in the fresh produce industry through drench application on the fruit surface within packhouses prior to packaging for both export and local markets. However, the effectiveness of essential oil nano-encapsulated coatings is highly dependent on the conditions of the experiment.. High-speed homogenisation could alter its concentration and lead to poor and unstable activity and an unknown release rate of the active ingredients. Further studies on the release rate of the predominant components in thyme oil and coating characterisation based on zeta potential, emulsion stability, polarity, and storage integrity should be investigated.

## Figures and Tables

**Table 1:** Impact of thyme nano emulsion essential oil, chitosan, standalone, their combination, and Sporekill^®^ on radial mycelial growth inhibition and nature of toxicity after 7 days of incubation at 25 °C against *C. gloeosporioides*.

Treatments	Concentrations		*Colletotrichum gloeosporioides*	
		Mycelia Growth (mm)	Inhibition (%)	Nature of Toxicity
**Nano-(T)-EO**	0.25%	38 ± 2 ^b^	38	Fungistatic
	0.5%	4.5 ± 0.5 ^g^	93	Fungistatic
	1%	0 ± 0.00 ^h^	100	Fungistatic
CS	0.2%	26.5 ± 0.5 ^c^	56.19	Fungistatic
	0.4%	23.5 ± 0.5 ^d^	61.15	Fungistatic
	0.6	12.5 ± 0.5 ^e^	79.33	Fungistatic
	0.8%	6 ± 1.00 ^f^	90.30	Fungistatic
	1%	5.4 ± 0.0 ^f^	91.20	Fungistatic
**Nano**-**(TEO-CS)**	1% + 0.8%	0 ± 0.00 ^h^	100	Fungistatic
Control		59 ± 0.03 ^a^	0	Fungistatic
Sporekill^®^	1.5%	0 ± 0.00 ^h^	100	Fungicidal

Values are expressed as mean ± SD. Mean values with different alphabetical letters in the same column are significantly different (*p* < 0.05).

## Data Availability

The original contributions presented in this study are included in the article. Further inquiries can be directed to the corresponding author.
